# Research on insulator defect detection algorithm of transmission line based on CenterNet

**DOI:** 10.1371/journal.pone.0255135

**Published:** 2021-07-29

**Authors:** Chunming Wu, Xin Ma, Xiangxu Kong, Haichao Zhu

**Affiliations:** 1 Key Laboratory of Modern Power System Simulation and Control & Renewable Energy Technology, Ministry of Education (Northeast Electric Power University), Jilin, China; 2 Department of Electrical Engineering, Northeast Electric Power University, Jilin, China; Fuzhou University, CHINA

## Abstract

The reliability of the insulator has directly affected the stable operation of electric power system. The detection of defective insulators has always been an important issue in smart grid systems. However, the traditional transmission line detection method has low accuracy and poor real-time performance. We present an insulator defect detection method based on CenterNet. In order to improve detection efficiency, we simplified the backbone network. In addition, an attention mechanism is utilized to suppress useless information and improve the accuracy of network detection. In image preprocessing, the blurring of some detected images results in the samples being discarded, so we use super-resolution reconstruction algorithm to reconstruct the blurred images to enhance the dataset. The results show that the AP of the proposed method reaches 96.16% and the reasoning speed reaches 30FPS under the test condition of NVIDIA GTX 1080 test conditions. Compared with Faster R-CNN, YOLOV3, RetinaNet and FSAF, the detection accuracy of proposed method is greatly improved, which fully proves the effectiveness of the proposed method.

## 1 Introduction

In modern society, the demand for electricity is increasing day by day, which poses a huge challenge to the inspection and maintenance of power grid. Daily inspection is a necessary means to meet this challenge and ensure the safe operation and stable operation of the power grid [1]. As an indispensable device in the power system, the self-destruction of the insulator will seriously endanger the safe operation of the power grid system. Therefore, it is particularly important to conduct state detection and fault diagnosis regularly. With the advancement of smart grid construction, more and more attention has been paid to UAV inspection. There are also more applications in power inspection work.

In recent years, the traditional insulator defect detection algorithms were mainly based on local features of images. Martinez et al. [2] proposed a method of transmission line tower detection and classification based on HOG feature and MLP neural network. Wang et al. [3] proposed to combine the shape, color and texture information of insulators for detection, which effectively reduces the influence of background texture and lighting. However, the above method is not effective in detecting occluded objects. Because it is difficult to extract complete features from the detected image to identify the insulator, it is difficult to achieve the expected accuracy. Since 2012, deep learning [4] received widely attention. There were two branches of object detection model: two-stage and one-stage detection model. The two-stage divides the whole process into two parts, with high detection accuracy, but it takes too long to achieve real-time detection effect. At present, many improved two-stage algorithms have been developed, for instance, R-CNN [5], Fast R-CNN [6], Faster R-CNN [7], R-FCN [8], etc. Compared with the two-stage, the one-stage can achieve end-to-end detection and has a faster detection speed, but its accuracy is reduced, mainly including: YOLO [9], SSD [10], YOLOv2 [11], YOLOv3 [12], CenterNet [13], etc.

Whether it is a two-stage detection model or a one-stage detection model, the information assistance of a priori box is usually needed to regress to the ground truth. However, the size and shape of defects change with the environment. Under the circumstances, it is hard to design suitable anchor frames, and the use of anchor boxes incurs more computational costs. Since Law and Deng proposed the Cornernet model without anchor boxes [14], some corresponding anchorless frame models have attracted widespread attention from scholars [13, 15–18]. Most of these detectors take key points, such as corners or centers, as positive samples to regress to the objects.

Therefore, on the basis of the above research, we propose a defect insulator detection algorithm based on WDSR and CenterNet, which uses ResNet50 as the backbone network. The WDSR algorithm is used to achieve super-resolution reconstruction. The network then identifies defective insulators. In addition, the generation of data set, the selection of evaluation indicators, the selection of network parameters and so on are deeply analyzed. Experimental results show that compared with YOLOv3 [12], RetinaNet [19], FSAF [18] and Faster R-CNN [7], the proposed method has more than 6.45% improvement in AP and more than 3.56% improvement in F1 score. It is proved that this method has better recognition effect on UAV detection image.

The following is the arrangement of other parts of the paper: the second section introduces the principle of the transmission line insulator defect detection and the construction of each part of the framework. The third section discusses the data set, experimental environment, result design, evaluation metrics, experimental design and result analysis. Finally, the fourth section summarizes the paper.

## 2 Method

**Fig 1:**
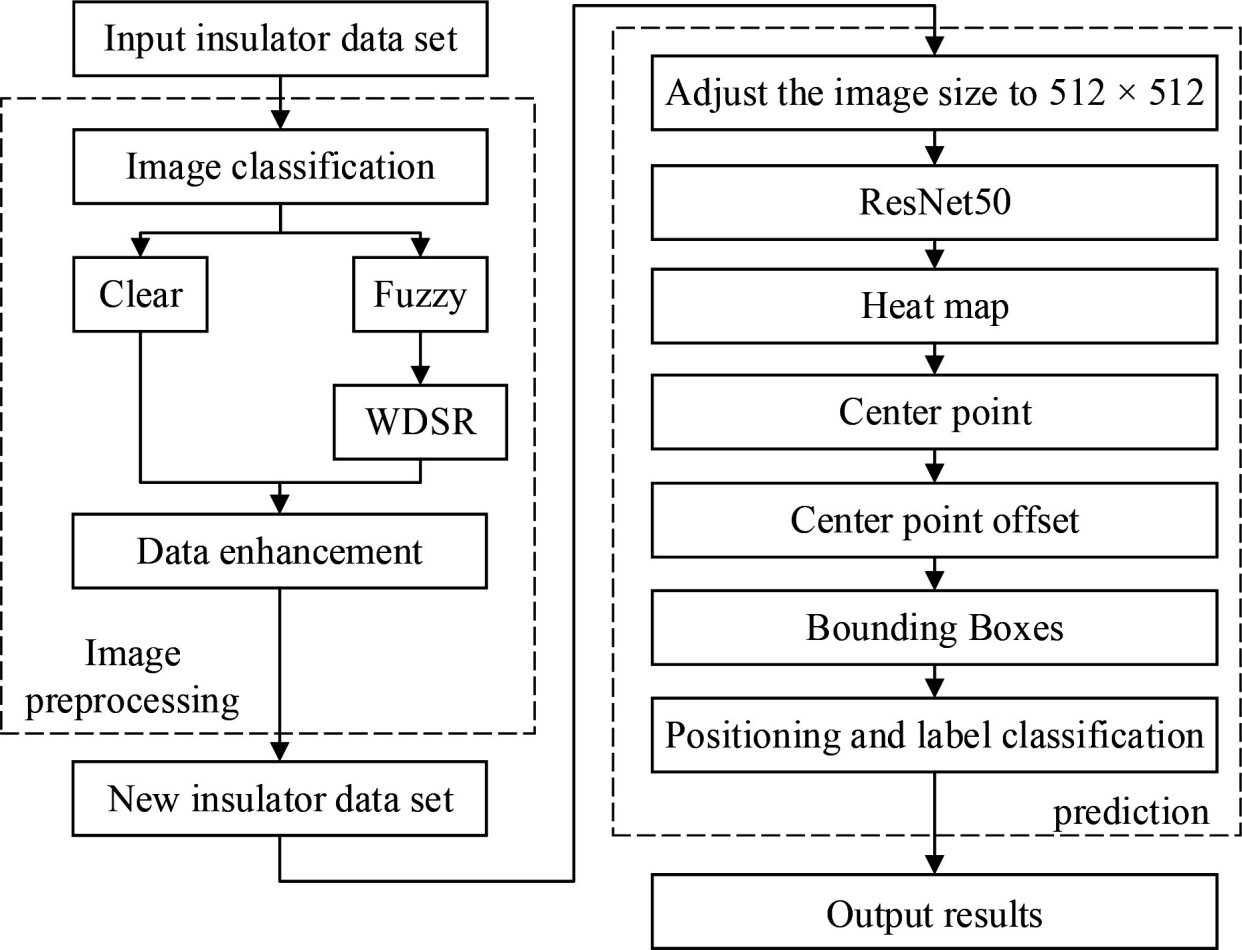
Flow chart of defect insulator detection.

This section introduces the defect detection framework for insulators of transmission lines. As shown in **Fig 1**. The defective insulator detection process includes image preprocessing and defective insulator detection.

The specific process of detection are as follows:

1. Divide the original UAV inspection image set into two categories: qualified image set and low-resolution blurred image set. In this paper, Laplace variance algorithm is used for image classification.
2. Super-resolution reconstruction via WDSR. The processed image is combined with the original image to obtain a suitable inspection image set through data enhancement.
3. Adjust the resolution of the new insulator image set to 512 × 512 resolution, and directly input it into the ResNet50 network to generate a heat map. The peak in the heat map is the center of the object.
4. The generation from point to bounding box goes through three parts: center point prediction, center point offset prediction and bounding box prediction.
5. Network output test results.

### 2.1 Backbone

In order to accelerate the optimization process and alleviate the gradient disappearance, a residual network is proposed in [20]. Later, many other experiments also proved that the residual network is very effective. ResNet50, the basic backbone network, is used in this experiment. However, limited by the amount of data in this experiment, the use of complex convolutional neural network may produce over fitting. Consequently, we improve the original CenterNet network with ResNet50 as the backbone.

**Fig 2:**
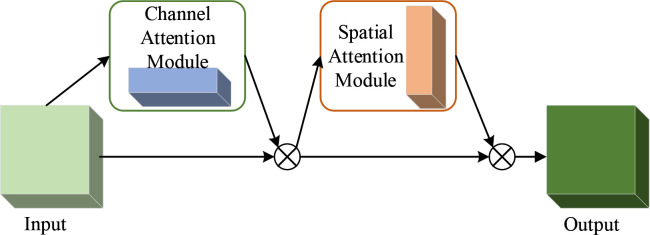
CBAM module structure.

In view of the characteristics of the insulator data set, such as large observation area, large amount of information, large difference in object size, few and independent large objects, and many and concentrated small objects, the attention mechanism is introduced. Attention mechanism can learn the features of insulator images well, suppress the non-object features, emphasize the instance information, suppress the background information, and improve the detection accuracy. In this paper, CBAM [21] is selected to help the model better select intermediate features. CBAM module is a universal and lightweight module, so it can be inserted into the convolution module of the whole network to achieve end-to-end synchronous training. We basically insert a 7×7 Attention module into the convolution module before the image is input into the ResNet50 backbone network. This module can improve the detection accuracy of small objects in the data set, because it helps the network to extract more key information in the image. The CBAM module is shown in **Fig 2**.

**Fig 3:**
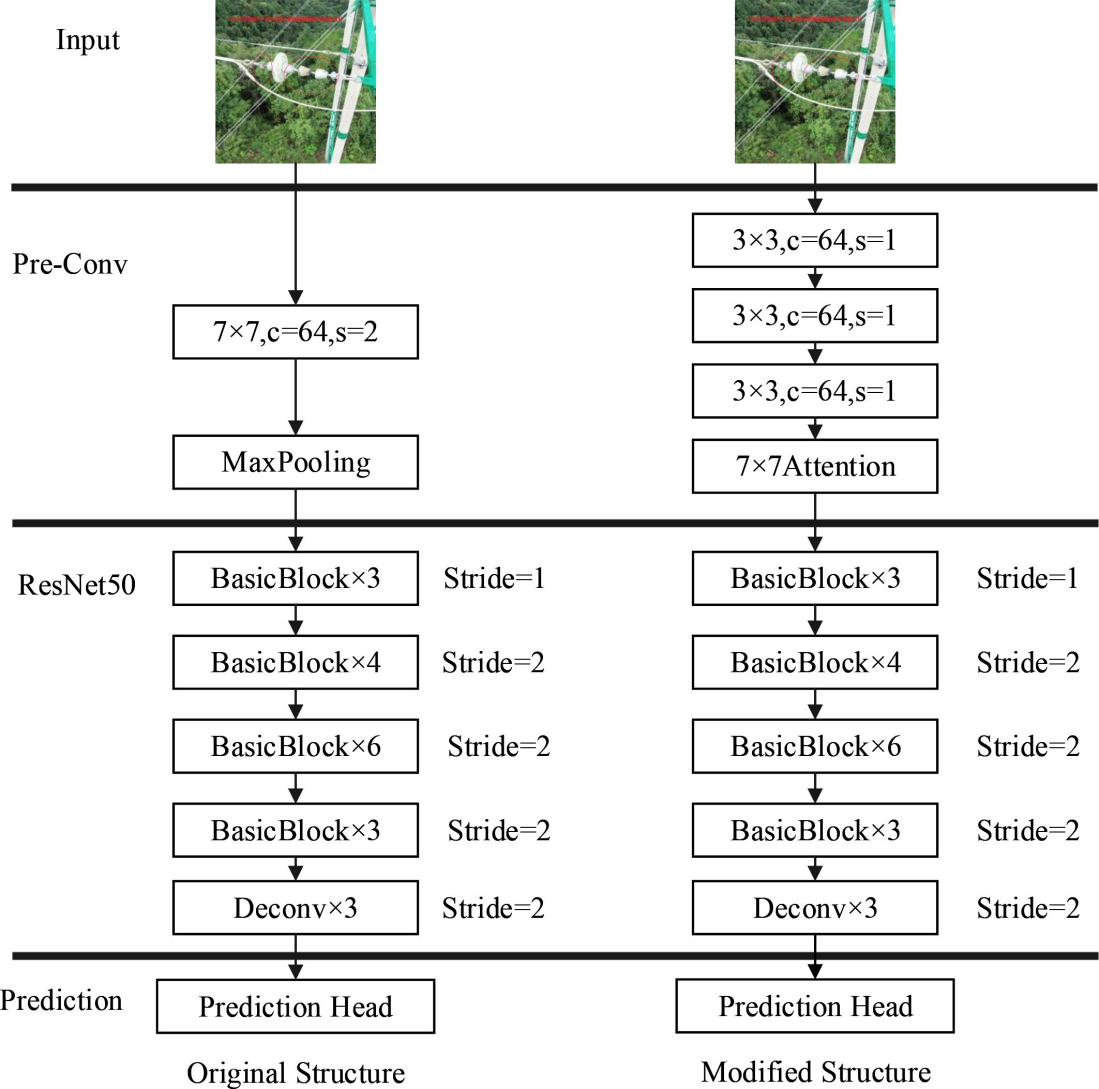
Comparison between the original model structure and the improved one.

According to [22], in the first convolutional layer, the down-sampling step may make the model performance worse, especially for small objects. In response to this situation, we substitute a 7 × 7 convolution layer (step 2) of the original network with three stacked 3 × 3 convolution layers (step 1). Among them, the channel of each 3×3 convolutional layer is set to 64, the purpose of which is to save computational cost. At the same time, we easily remove the pooling layer. The comparison between the original model structure and the improved one is shown in **Fig 3**.

### 2.2 Detecting centers

**Fig 4:**
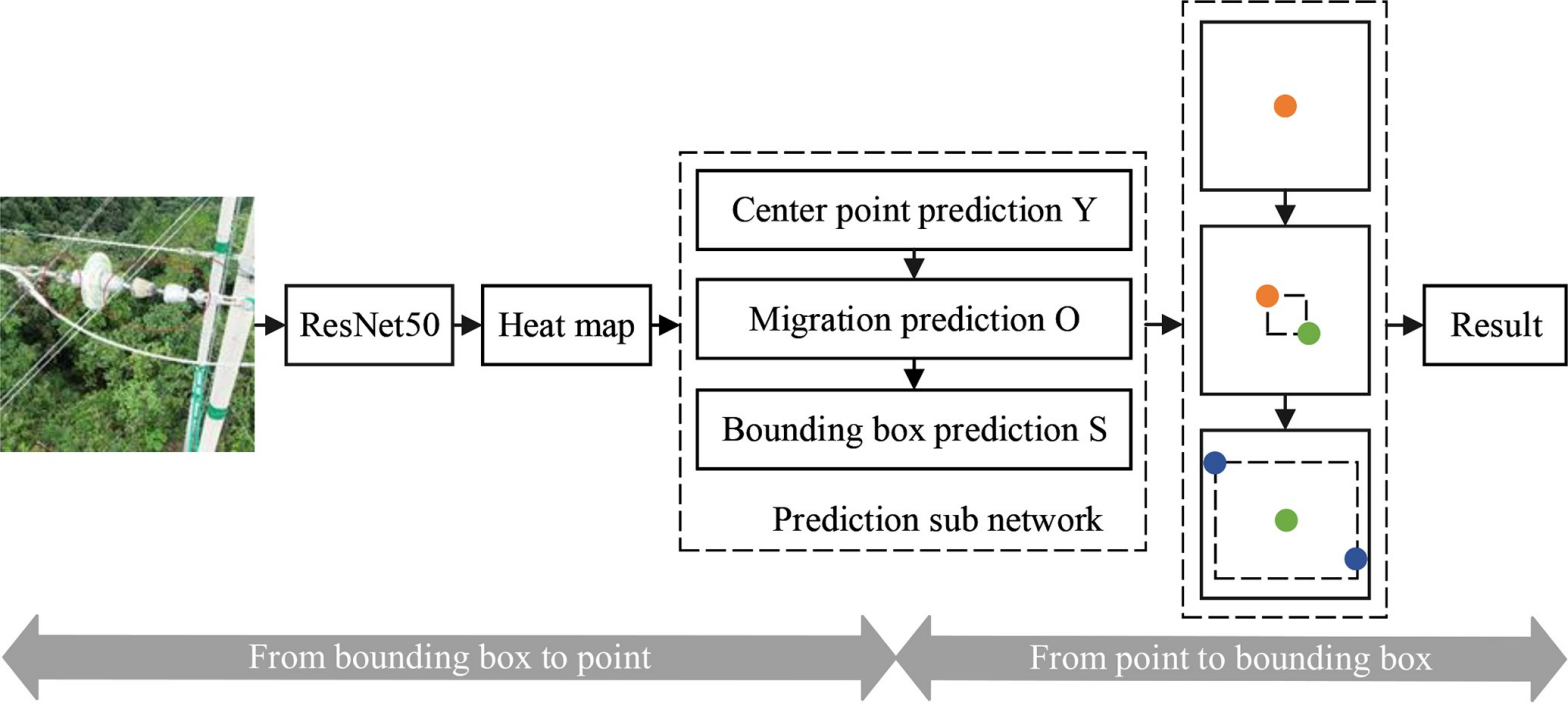
Network architecture of CenterNet.

The process from the bounding box to point is shown in **Fig 4**. The labeled image is put into the feature extraction network to obtain the output feature map. Then the key point prediction branch Y, the center point deviation branch O and the object size branch S share the same feature extraction network for training respectively.

We use the center heatmap to classify and locate the defective insulators, but in order to avoid the influence on the foreground prediction score, the background channel is not used. The resolution of the image is reduced by 4 times through ResNet50, and then the feature map is up-sampled and restored to its original size. In short, the resolution of the input image is equal to that of the center heatmap. Assuming that the size of the input image is W×H×3, the size of the corresponding heatmap is C×W×H, where the C channel represents category C. Since we only detect insulator self-explosion, C is set to 1. For a defective insulator string, only the center of its bounding box is positive, with a value of 1. All other positions are negative with a value of 0. However, this can produce a serious imbalance between positive and negative samples, which can reduce the generalization ability of the model. Therefore, we use Gaussian functions [13, 14] to process the points around the center and reduce their contribution to the loss. The function is given by:

$$Y_{xyc}=exp\left(-\frac{{\left(x-\bar{P_{x}}\right)}^{2}+{\left(y-\bar{P_{y}}\right)}^{2}}{2\sigma_{P}^{2}}\right)$$ (1)

where $\bar{P_{x}}$ and $\bar{P_{y}}$ is the center point coordinate, σ is the variance. The value of σ depends on the radius r of the region around the center. The parameter r is determined by the method in [14], that is, the IoU value of the prediction box and the ground truth reaches at least 0.3, so σ = 1/3r is set.

**Fig 5:**
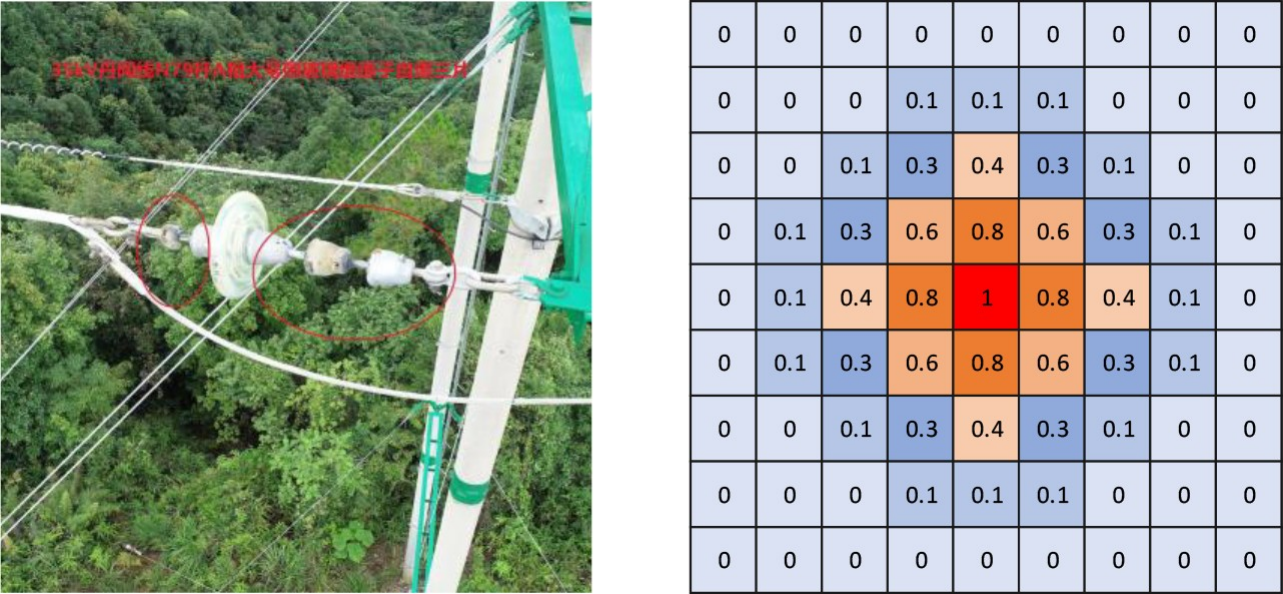
The center heatmap icon. (a) Original image, (b) Gaussian label.

The center heatmap icon is shown in **Fig 5**.

Here, the training loss refers to [14] and it is derived from Focal Loss [19], which is defined as:

$$L_{cls}=\frac{-1}{N}\sum_{c=1}^{C}\sum_{i=1}^{H}\sum_{j=1}^{W}\{\begin{array}{l} \;{\left(1-p_{cij}\right)}^{\alpha }\;\log(p_{cij})\;ify_{cij}=1\\ {\left(1-y_{cij}\right)}^{\beta }{\left(p_{cij}\right)}^{\alpha }\;\log(1-p_{cij})\;otherwise \end{array}$$ (2)

where N is the number of defective insulator pieces. *p_cij_* is the predicted score of class C at point (i, j), and is the corresponding label. α and β are hyperparameters. And β is used to control the weight of points around the positive sample. Set α = 2 and β = 4.

### 2.3 Bounding boxes regression

We return to the bounding box through the center point (positive point). Assume defect i has a label of (*x*_*i*min_,*y*_*i*min_,*x*_*i*max_,*y*_*i*max_). So the bounding box can be expressed as *box_i_* = (*x*_*i*max_−*x*_*i*min_,*y*_*i*max_−*y*_*i*min_). Then the training loss we use is L1 loss [6]:

$$L_{reg}=\frac{1}{N}\sum_{i=1}^{N}smooth_{L_{1}}(box_{i}^{*}-box_{i})$$ (3)

$$smooth_{L_{1}}(x)=\{\begin{array}{c} 0.5x^{2}\;if\left|x\right|<1\\ \left|x\right|-0.5\;otherwise \end{array}$$ (4)

where $box_{i}^{*}$ is the predicted value of the bounding box.

### 2.4 Implementation details

The resolution of the image in the data set needs to be adjusted to 512 × 512. In order to improve the sample imbalance, we add more negative samples to some images. In addition, random clipping, flipping and color dithering are used in the data enhancement part, which can alleviate the problem of overfitting. We also use the Adam [23] optimizer. The sum of the losses of the two branches is the total loss.

$$Loss=\alpha L_{cls}+\beta L_{reg}$$ (5)

where α = 1.0, which is the weight of *L_cls_*, and β = 0.1, which is the weight of *L_reg_*. Because the model structure is relatively simple, so only one GPU can train the model. We can use a batch size of 16, and train the whole network for 50 epochs with initial learning rate 1.5×10−4. Among them, the learning rate is reduced to 2.5×10−5 after 30 epochs.

## 3. Experiment

### 3.1 Dataset and compared methods

**Fig 6:**
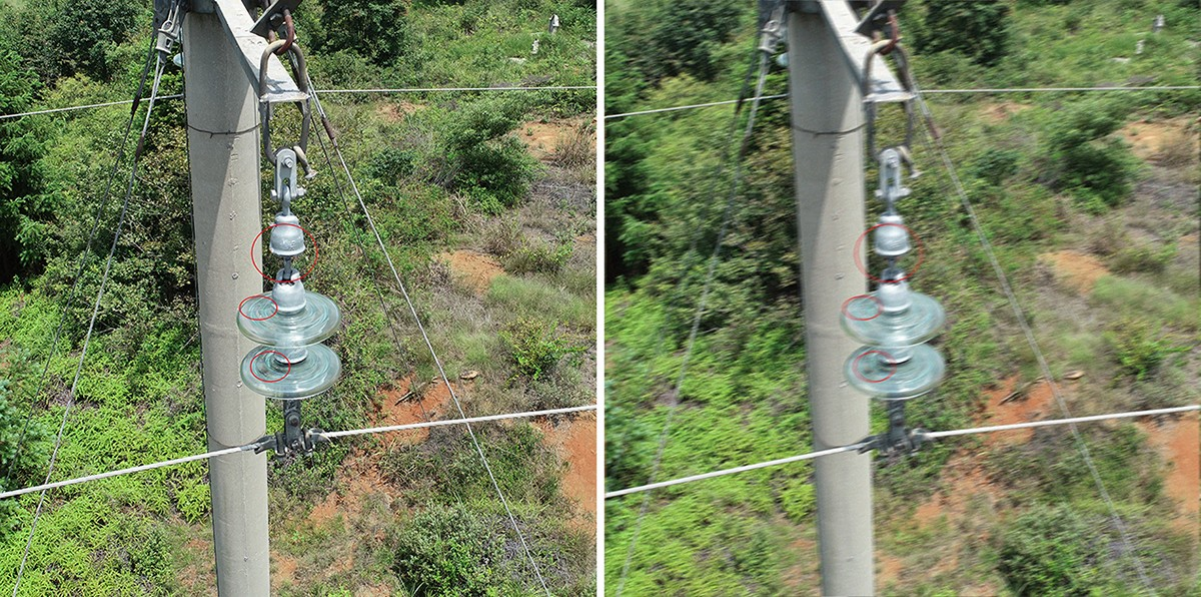
The insulator image after motion blur. (a) The original image, (b) The image after motion blur.

When preparing the training data set for WDSE, we follow the training methods in [24] and [25]. At the same time, the clear image is processed by the motion blur method, where the blur radius is set as 7. Finally, the blurred image and the corresponding clear image are combined into a training pair as a training set. The insulator image after motion blur is shown in **Fig 6**.

**Fig 7:**
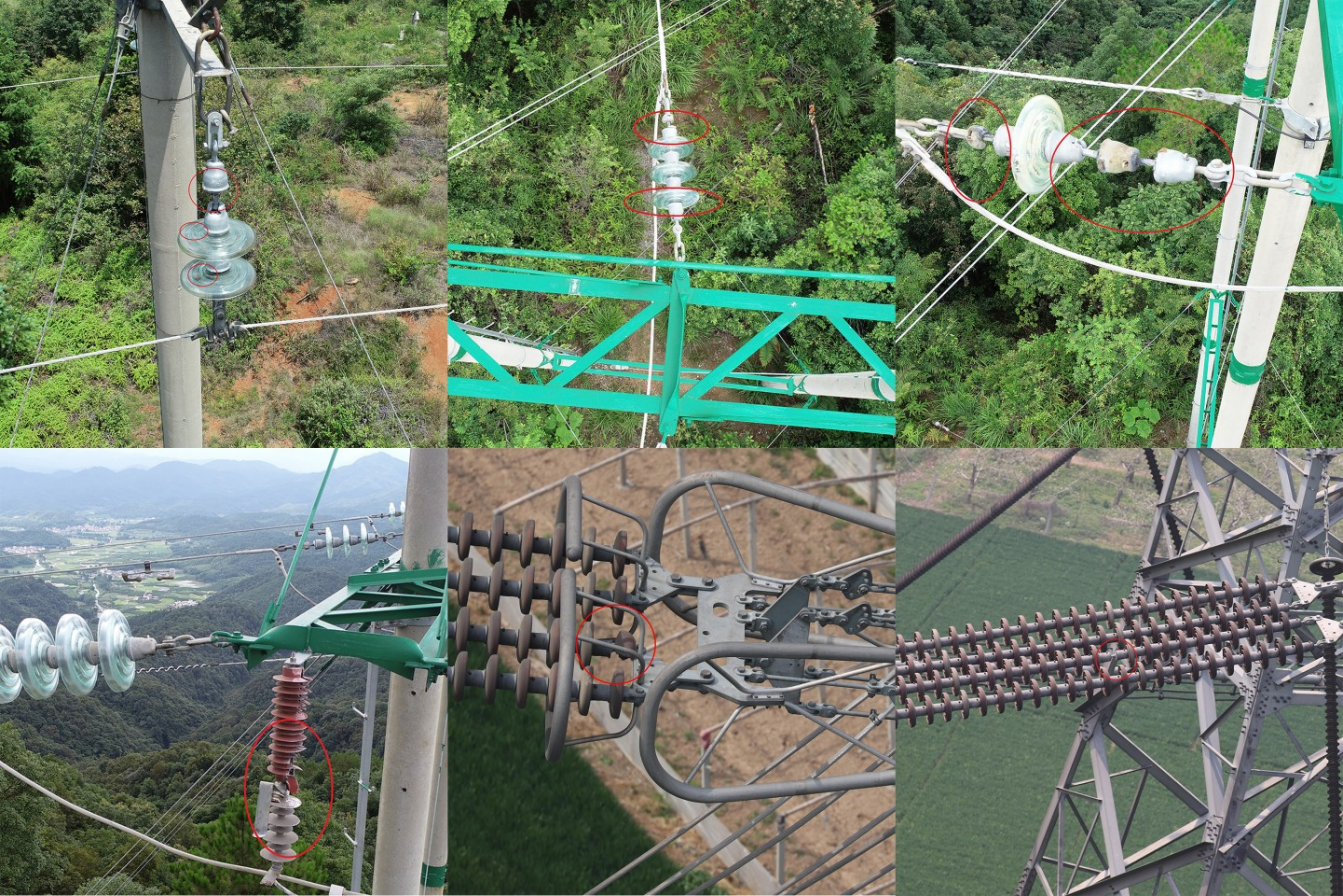
Partial image set of insulators.

The data set of insulator detection part in this experiment consists of two parts: 1507 network images and 931 UAV aerial images. The UAV images used in this experiment are all taken from the inspection of a power company in Guangdong Province. The training set and the test set consist of 1958 and 480 images respectively. A partial image of the dataset is shown in **Fig 7**.

**Fig 8:**
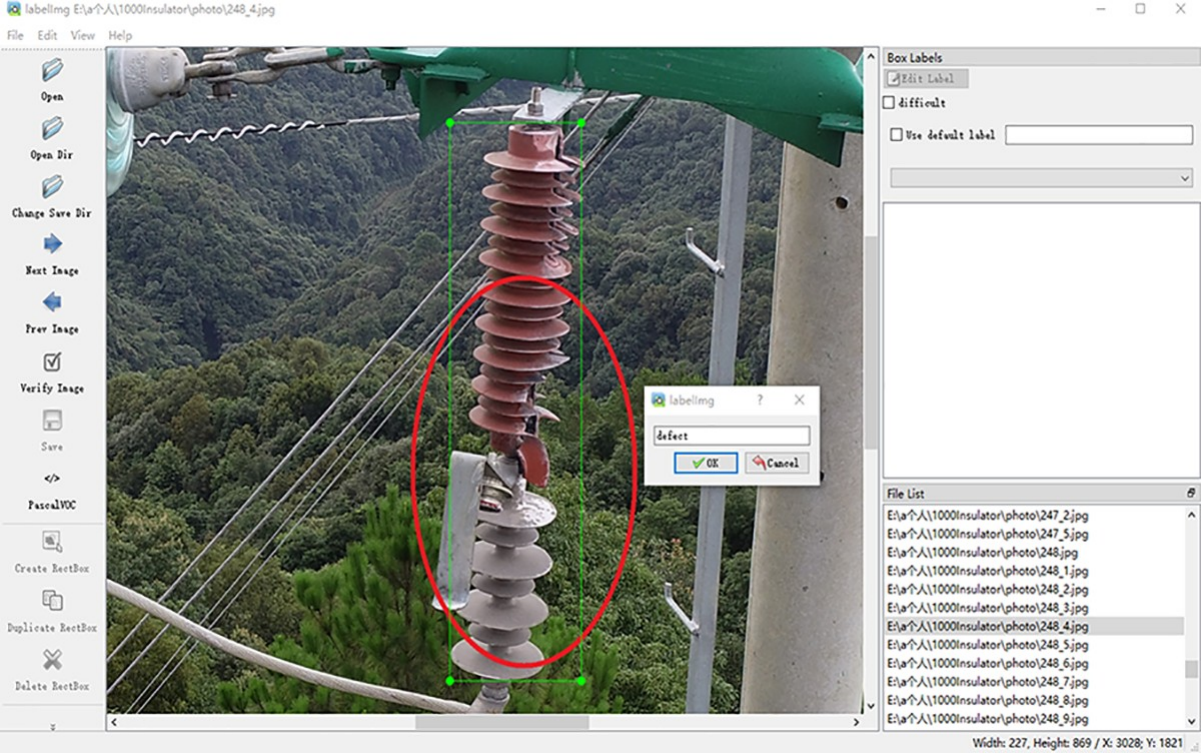
The labeling diagram of LabelImg.

For the convenience of sample management and index, the samples are named XXX_ x. Jpg format. The labeling diagram of LabelImg is shown in **Fig 8**.

In this paper, the experimental settings used for comparison are as follows: YOLOv3 [12], RetinaNet [19] and FSAF [18] are selected as the one-stage detection. The two-stage detection uses Faster R-CNN [7].

The detection effect of YOLOv3 on small objects is better, because it uses feature pyramid information for detection. In order to ensure network performance, this experiment chooses pre-trained darknet-53 as the backbone.

RetinaNet improves the accuracy of two-stage detection because it makes use of Focal Loss to reduce the weight of a great quantity of simple negative samples in training. This method requires the input image to be 640×640, and this experiment uses ResNet50 as the backbone.

FSAF has two branches: anchor-based branch and anchor-free branch. Each object dynamically selects the best feature layer. After the selection is made, the anchor-based method is used for subsequent classification and position regress. The basic backbone selected in this experiment is also ResNet50.

The Faster R-CNN detector is very popular due to its high detection accuracy. The method of Faster R-CNN to obtain candidate boxes is the RPN (Region Proposal Network), and then the detector classifies these regions. Both parts share ResNet50 as the backbone.

The above comparison experiments using ResNet50 as the backbone, the backbones are all pre-trained on the MS COCO data set. In 200 epochs of training, we use the Adam [23] optimizer for all methods. Set the initial learning rate to 10–4 in the first 90 epochs, drop to 10–5 in 90 epochs, and 10–6 in 150 epochs. To ensure the comparability of the results, both training and testing are performed on our data set.

### 3.2 Evaluation metrics and detection results

Precision, recall and PRC (precision recall curve) [26] are used to measure the performance of the above methods. The calculation methods of recall and precision are as follows:

$$Recall=\frac{TP}{TP+FN}$$ (6)

$$Precision=\frac{TP}{TP+FP}$$ (7)

where TPs, FPs and FNs represent true positive, false positive and false negative respectively.

AP (average precision), F1 score and FPS (frames per second) are also used as evaluation indexes. The F1 score represents the golden ratio of precision and recall, that is, the weighted harmonic average of precision and recall. FPS is detected by using a camera to simulate the video stream obtained by the UAV under the environment of a single NVIDIA GTX1080 graphics card in the micro-star deep learning workstation in this paper. The average detection time of 100 images is calculated to get the inference speed index of this model. The calculation method is as follows:

$$AP=\frac{\sum Precision}{Num(Total\;Objects)}$$ (8)

$$F1=2\times \frac{Precision\times Recall}{Precision+Recall}$$ (9)

**Fig 9:**
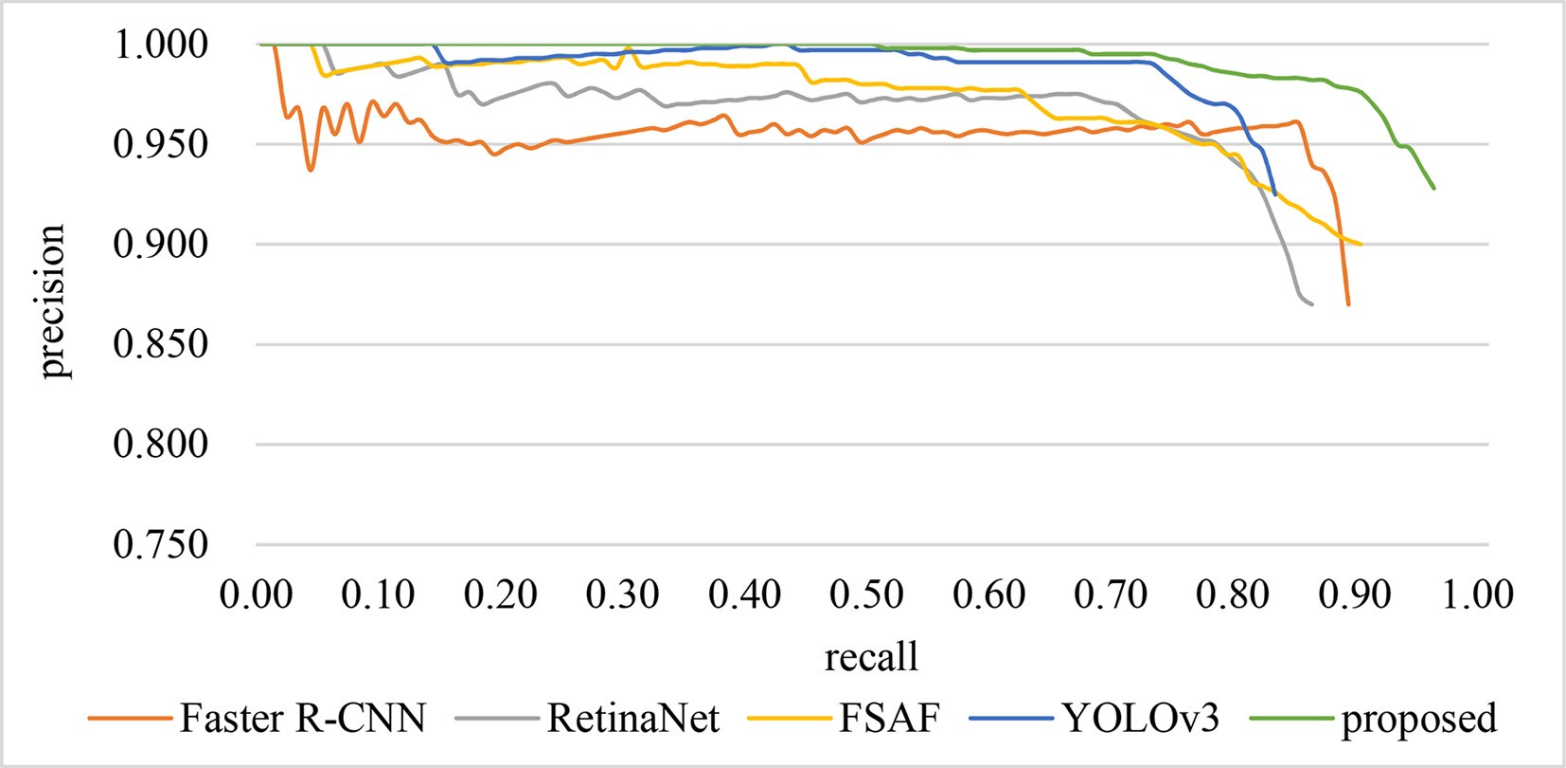
PRC of different methods.

**Fig 10:**
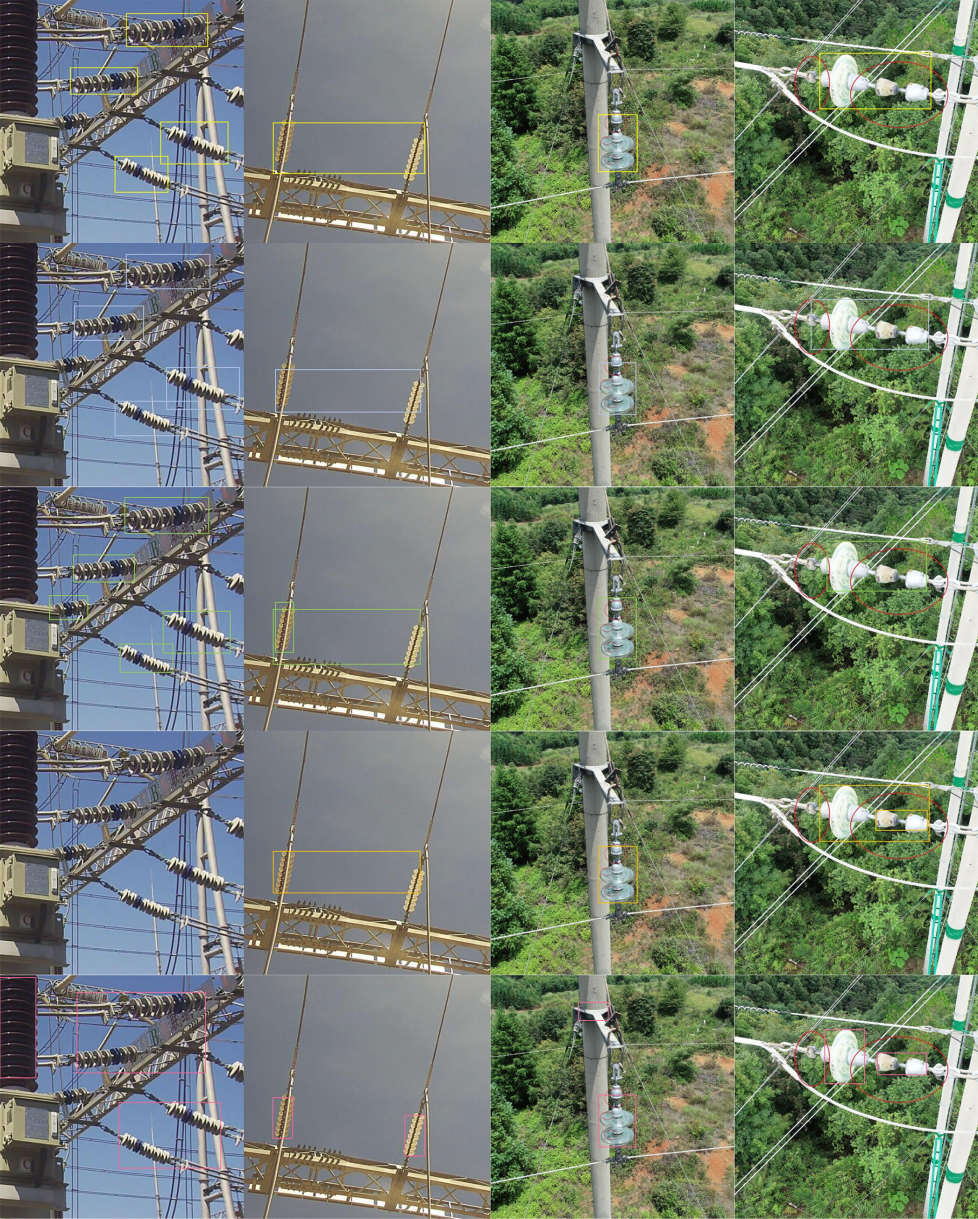
The visual effects of different methods of testing results. From top to bottom are the visual results of Faster R-CNN, YOLOv3, RetinaNet, FSAF and our proposed method.

The PRCs of all networks are shown in **Fig 9**. Our results on the defect data are shown in Table 1. Qualitative comparisons with other methods are shown in **Fig 10**.

**Table 1:** Main detection results.

Method	P(%)	R(%)	AP(%)	F1(%)	FPS
Faster R-CNN	90.51	89.94	86.96	90.22	5
YOLOv3	93.24	83.81	83.27	87.92	**57**
RetinaNet	87.90	88.19	86.28	88.04	17
FSAF	91.55	91.36	89.71	91.45	21
Ours CenterNet	90.11	95.48	95.48	92.72	33
Ours CenterNet+CBMA	**94.02**	**96.02**	**96.16**	**95.01**	30

It can be clearly concluded from the figure that our methods perform well in terms of accuracy and recall. Specifically, the accuracy rate is only a little higher than other networks, but the recall rate is far higher than other recall rates. Although Faster R-CNN is a two-stage model, its recall is surprisingly good. This is due to the fact that the RPN generates a suitable anchor box for the candidate insulators. The accuracy of YOLOv3 is second only to the method we proposed, but the price is that its recall is the lowest among the above methods. Through the visual output of YOLOv3, it can be seen that the reason leading to the lowest recall rate is that some insulators are selected through the bounding box, but the size and position of the bounding box are not accurate enough. RetinaNet is exactly the opposite of YOLOv3. It has a higher recall but a lower accuracy. Comprehensive comparison, the performance of FSAF is the most stable.

Table 1 shows that the improved method with CBMA obtains the best AP and F1 score. By improving ResNet50, the AP value of the network reaches 95.48% and the F1 score reaches 92.72%. Moreover, FPS is also fast, ranking second in the above methods, and can be detected in real-time. In addition, the AP and F1 score reaches 96.16% and 95% respectively after adding the attention mechanism, which is the highest score among the above methods, which proves the effectiveness of this method. Among all networks compared, the AP of FSAF is closest to our effect. In comparison, FASF performs well in the detection of small defective insulators, and the other three methods perform well in the detection of large defective insulators, but poorly at detecting small defective insulators and insulators with incomplete shapes.

It can be seen from Fig 10 that compared with other methods, our methods are more robust to the detection of defective insulators. This is reflected in the detection effect of similar objects, side-by-side objects and multi-scale objects.

## 4. Conclusion

Based on ResNet50, we improve the original CenterNet, simplify the whole backbone network and realize the detection of insulator piece falling off. The experiment shows that the detection result of the insulator sheet falling off reaches AP (96.16). It is verified that the effect of the improved CenterNet is excellent, it can also be detected in real-time, and has special practical significance to improve the power detection technology.

UAV line inspection is the general trend of electric power inspection. Our next step in this field is to establish a unified insulator database and accurately distinguish the fault types, including lightning stroke, icing, self-explosion, etc. In this way, not only the fault detection of insulators can be realized, but also their defects can be classified.
